# Supplementary material for: DNA Deamination Is Required for Human APOBEC3A-Driven Hepatocellular Carcinoma In Vivo
Source: Int J Mol Sci. 2023 May 26;24(11):9305. doi: 10.3390/ijms24119305 (PMC10253583; doi:10.3390/ijms24119305)
Supplement: Supplementary file 1 [file ijms-24-09305-s001.zip › ijms-2397065-supplementary.pdf]

Supplementary Materials

Supplementary Information: Supplementary Figures S1-S3

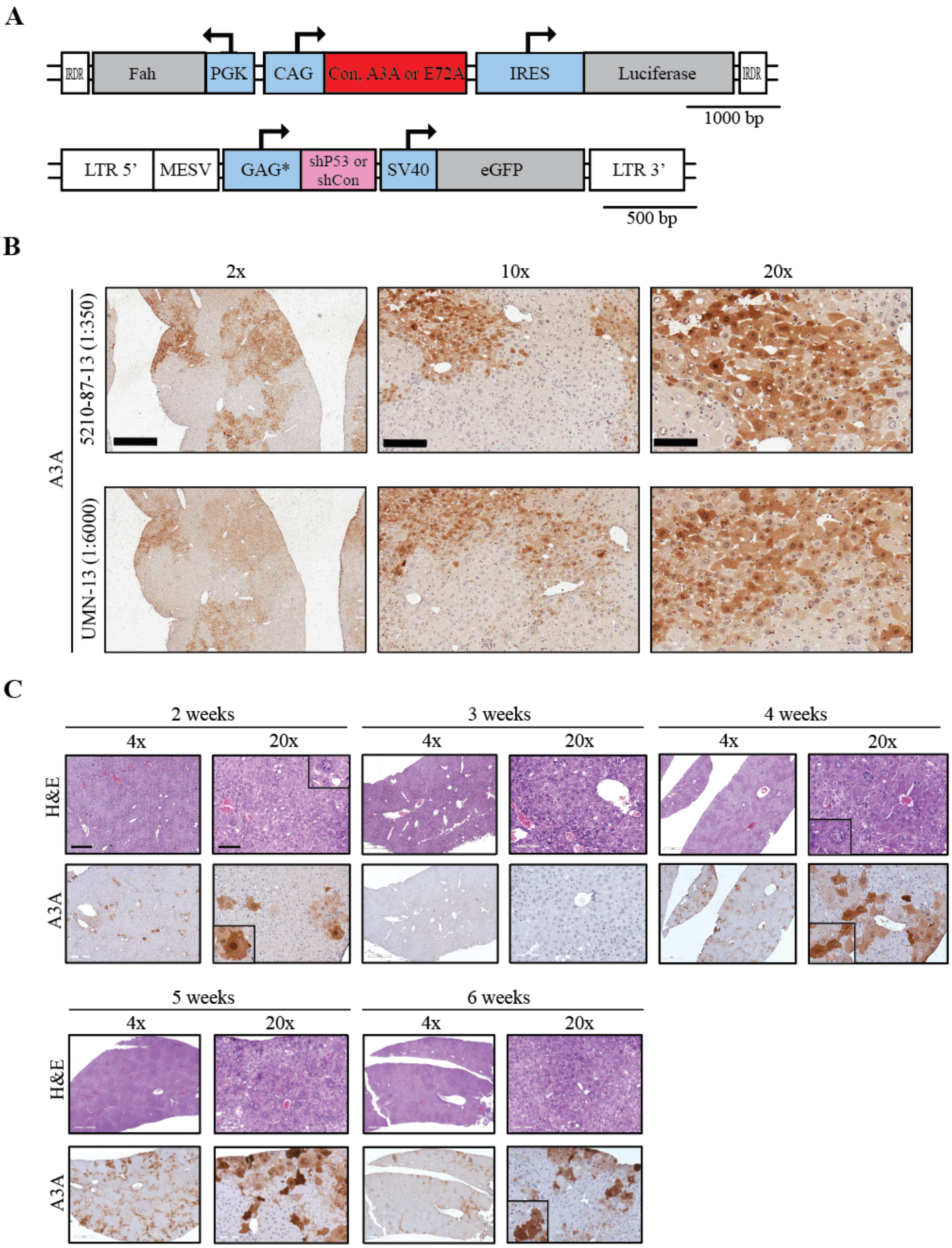
A
IRDR
Fah
PGK
CAG
Con. A3A or E72A
IRES
Luciferase
IRDR
1000 bp
LTR 5'
MESV
GAG*
shP53 or shCon
SV40
eGFP
LTR 3'
500 bp
B
2x
10x
20x
A3A
5210-87-13 (1:350)
UMN-13 (1:6000)
C
2 weeks
3 weeks
4 weeks
4x
20x
H&E
A3A
5 weeks
6 weeks

Supplementary Figure S1. Human A3A expression in murine hepatocytes.

(A) Schematic representation of Fah-restorative and shRNA constructs used for hydrodynamic injections in Figure 1. Scale bar, 1000 bp and 500 bp, respectively.

(B) Comparative IHC analysis of consecutive sections of A3A expressing livers stained with α-A3A monoclonal antibodies, 5210-87-13 or UMN-13, at 1:350 or 1:6,000 dilutions, respectively. Scale bar, 1mm, 200 µm, and 100 µm for 2x, 10x, and 20x magnification, respectively.

(C) Representative H&E and A3A IHC (UMN-13 mAb) photomicrographs of A3A livers harvested 2-6 weeks p.i. Scale bar, 1 mm and 100 µm for 4x and 20x magnification, respectively, and inset images are magnified 4-fold.

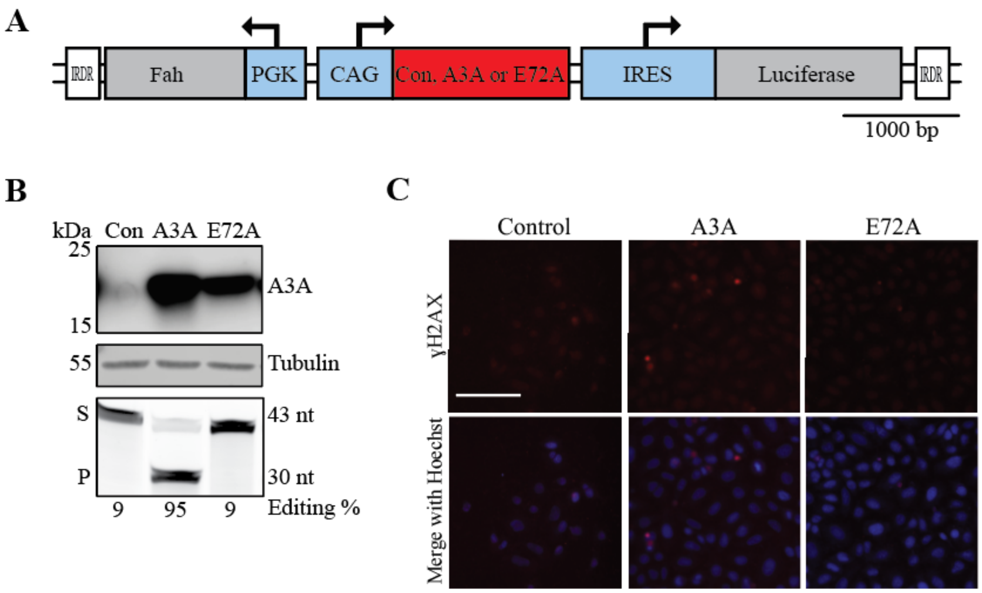

Supplementary Figure S2. The catalytic activity of A3A induces genomic damage.

(A) Schematic representation of E72A construct used for hydrodynamic injections. Scale bar, 1000 basepairs.

(B) Immunoblot (upper panels; 5210-87-13 mAb) and DNA deaminase activity assay (lower panel) of whole cell lysates of HeLa cells transfected with constructs containing A3A, E72A or control. Control (Con); substrate (S); product (P); nucleotides (nt). DNA deaminase activity quantification of band volume intensities (ratio of substrate to product) by densitometry reported below as Editing %.

(C) Immunofluorescence analysis of transfected HeLa cells stained for γ-H2AX and counterstained for DNA with Hoescht. Scale bar, 100 μm.

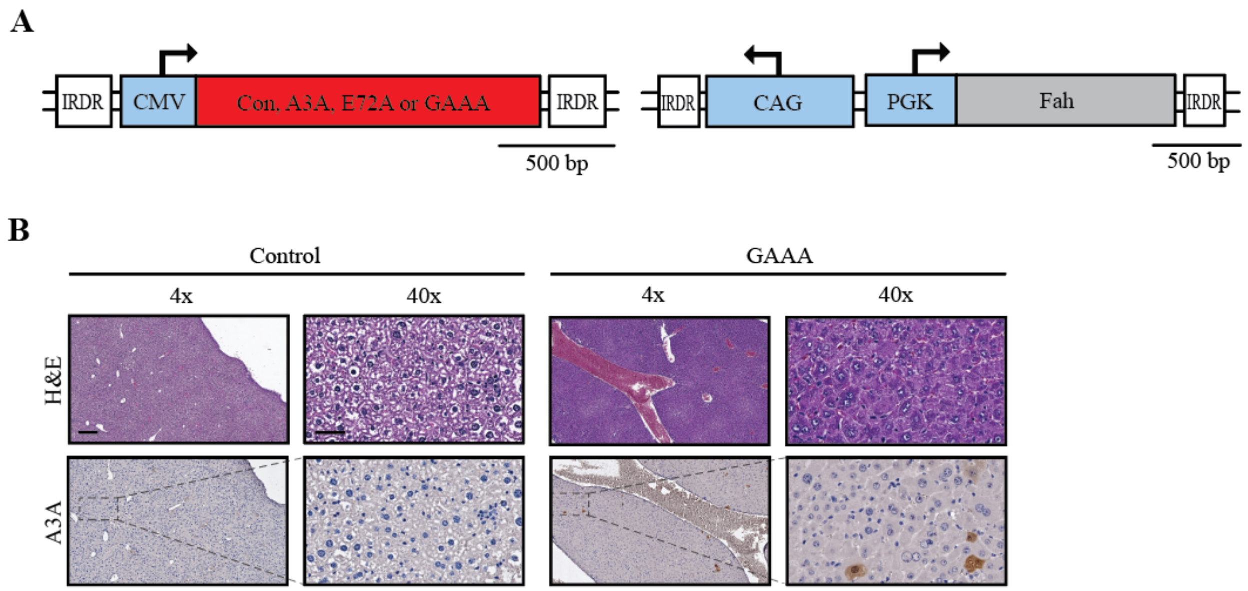

Supplementary Figure S3. A3A-GAAA mutant expression in hepatocytes.

(A) Schematic representations of constructs used for all experiments in Figure 4. Scale bars, 500 bp.

(B) H&E and IHC (UMN-13 mAb) of representative livers from the Control and GAAA cohorts harvested 72 hours p.i. Scale bar, 500 μm or 50 μm for 4x and 40x magnification, respectively.
